# Plastome variation and phylogenetic relationships of *Salsola* species in China

**DOI:** 10.3389/fpls.2026.1919433

**Published:** 2026-07-28

**Authors:** Yiheng Wang, Haiwen Li, Jiatong Sun, Bo Mu, Xin Meng, Tao Dong, Lu Wang, Yanlei Liu, Yunjuan Zuo, Shuo Shi, Zhili Suo

**Affiliations:** 1 State Key Laboratory for Quality Ensurance and Sustainable Use of Dao-di Herbs, National Resource Center for Chinese Materia Medica, Academy of Chinese Medical Sciences, Beijing, China; 2 College of Life Sciences and Technology, Tarim University, Alar, China; 3 School of Landscape and Ecological Engineering, Hebei University of Engineering, Handan, China; 4 College of Life Science, Hebei Normal University, Shijiazhuang, China; 5 Center for Integrative Conservation, Xishuangbanna Tropical Botanical Garden, Chinese Academy of Sciences, Xishuangbanna, China; 6 State Key Laboratory of Systematic and Evolutionary Botany, Institute of Botany, Chinese Academy of Sciences, Beijing, China

**Keywords:** medicinal utilization, phylogeny, plastome, *Salsola*, species identification

## Abstract

The genus *Salsola* (*Amaranthaceae*) comprises approximately 130 species distributed across Asia, Africa, and Europe, many of which possess important medicinal, horticultural, and ecological significance. Known for its remarkable ecological plasticity, *Salsola* species thrive in arid, semi-arid, and saline-alkaline environments, serving as key components of desert plant communities. However, extreme environmental pressures have driven convergent evolution, resulting in morphological similarities that obscure phylogenetic relationships. While this structural complexity offers significant horticultural potential, the genus’s evolutionary branching remains poorly understood. In this study, we analyzed complete plastomes of 14 well-defined *Salsola* species to resolve these primary phylogenetic lineages. The results show that *Salsola* plastomes range from 149,653 to 152,969 bp in length and harbor 130 annotated genes. These sequences exhibit high synteny, with inverted repeat (IR) regions showing greater conservation than single-copy regions. Our phylogenetic analysis resolved the genus into two basal clades. One clade predominantly consists of annual herbs (e.g., *S. brachiata*, *S. collina*, and *S. ruthenica*), while the other includes the shrub *S. arbuscula* and semi-shrubs such as *S. orientalis*, *S. passerina*, and *S. abrotanoides*. From the perspective of complete plastome data, our results appear to support *Salsola* as a well-supported monophyletic lineage. Nevertheless, this inference should be treated with caution because it is based solely on plastome evidence; broader taxon sampling and nuclear genomic data will be needed to further test this conclusion. By providing complete plastome resources for representative *Salsola* species, this study offers a genomic foundation for future taxonomic clarification, species identification, germplasm conservation, medicinal and horticultural utilization of the genus.

## 1 Introduction

The genus *Salsola* (Amaranthaceae) comprises approximately 130 species distributed globally, with primary concentrations in Asia, Africa, and Europe. The region from the Mediterranean to Central Asia represents the diversity hotspot and core distribution range for the genus, although several species have extended into Oceania and the Americas, reflecting their remarkable adaptability across continents (Akhani et al., 2014; Hanif et al., 2018; Murshid et al., 2022). China hosts 36 species and one variety, representing nearly one-third of the global total (Editorial Committee of the Flora of China CAoS, 1990). Species of *Salsola* are highly adapted to arid, semi-arid, and saline-alkaline environments, where they often dominate desert and saline ecosystems and play important ecological roles (Johnson, 1998; Benderdouche et al., 2003; Hu et al., 2012; Yildiztugay et al., 2014; Centofanti and Bañuelos, 2015; Hanif et al., 2017). In addition to their ecological importance, several species possess considerable medicinal, nutritional, and forage value, highlighting the economic significance of the genus (Tundis et al., 2009; Altay and Ozturk, 2020; Iannuzzi et al., 2020; Mohammed et al., 2021; ElNaggar et al., 2022; Golovchenko et al., 2022; Soliman et al., 2022; Hameed et al., 2023; Wang et al., 2024). Accurate taxonomic classification provides an essential foundation for the effective conservation, utilization, and genetic improvement of *Salsola* resources. Since Linnaeus designated *Salsola kali* L. as the type species in 1753, the genus has attracted considerable taxonomic attention. Although the taxonomy of *Salsola* in China has been continuously revised, species delimitation and infrageneric classification remain insufficiently resolved (Forbes and Hemsley, 1888; Editorial Committee of the Flora of China CAoS, 1990).

Owing to extensive morphological convergence associated with adaptation to desert and saline environments, species delimitation and generic boundaries within *Salsola* have remained problematic. The development of molecular markers since the 1970s has promoted advances in the taxonomy and systematics of the genus (Gaskin et al., 2006; Ryan et al., 2007; Hrusa and Gaskin, 2008; Grozeva et al., 2018; Almerekova et al., 2024; Li et al., 2025; Wang et al., 2025). Comprehensive molecular phylogenetic studies based on nuclear and plastid DNA markers demonstrated that *Salsola* sensu lato is polyphyletic and comprises multiple distinct evolutionary lineages. Consequently, several genera, such as *Caroxylon*, *Kali*, *Kaviria*, *Turania*, *Pyankovia*, and *Xylosalsola*, were resurrected or newly established. Subsequent studies further supported the segregation of some lineages and even described additional genera, such as *Akhania*. Nevertheless, phylogenetic relationships among many species remain insufficiently resolved, and the taxonomic treatment of *Salsola* and its allied genera continues to be debated (Akhani et al., 2007; Wen et al., 2010; Sukhorukov et al., 2022). However, most previous phylogenetic studies were based on a limited number of molecular markers, and comprehensive plastome-scale evidence remains scarce for many species. Therefore, whether the currently recognized segregate genera truly represent independent evolutionary lineages requires further evaluation using plastome data.

Compared with other cytoplasmic genomes, the plastome is characterized by its relatively conserved structure, compact size, and uniparental inheritance, making it an important molecular resource for plant phylogenetic and evolutionary studies (Wolfe et al., 1987; Keeling, 2004). Although conventional DNA barcoding has been widely used in plant taxonomy, its ability to resolve closely related species is often limited (Hebert et al., 2003; Kress et al., 2005; Group1 CPW et al., 2009; Li et al., 2015; Cheng et al., 2016; Hobern and Hebert, 2019; Liu et al., 2024). With the rapid development of high-throughput sequencing technologies and declining sequencing costs, complete plastome sequencing has become an effective approach for comparative genomic and phylogenetic studies (Dong et al., 2025; Wu et al., 2026). Researchers now advocate for the use of the complete plastome as a “super-barcode” to achieve definitive species identification (Li et al., 2015; Zhang et al., 2021; Liu et al., 2024).

As pioneer plants in desert ecosystems, *Salsola* species are of considerable ecological, medicinal, and economic importance in Northwest China. However, the relatively conserved floral and vegetative morphology of many species poses challenges for traditional taxonomic identification and phylogenetic inference, particularly among closely related taxa (Toderich et al., 2010). Although previous molecular studies have improved our understanding of the phylogeny of *Salsola*, plastome-scale evidence remains limited for many Chinese species. In this study, we performed comparative analyses of plastomes from representative Chinese *Salsola* species to characterize plastome variation, reconstruct intrageneric phylogenetic relationships, identify highly variable regions, and evaluate the potential utility of plastome data for species delimitation and identification.

## 2 Materials and methods

### 2.1 Materials

At present, there are 36 species and one variety of *Salsola* documented in China. In this study, 14 *Salsola* species were selected as sequencing samples, namely *S. abrotanoides*, *S. arbuscula*, *S. heptapotamica*, *S. pellucida*, *S. collina*, *S. passerinum*, *S. implicata*, *S. orientalis*, *S. nitraria*, *S. micranthera*, *S. subcrassa*, *S. affinis*, *S. bracchita*, and *S. tragus*. The research materials were sourced from the Herbarium of the Institute of Botany, Chinese Academy of Sciences (PE). In addition, we incorporated complete plastome sequences obtained from the NCBI public database, including three *Caroxylon* species (one sample per species), one *Pyankovia* species (one sample), and one *Oreosalsola* species (one sample) (**Table 1**).

**Table 1:** Samples and plastomes used in this study.

Original Latin name	New Latin name	Original number	Accession number	Plastome length	Identifier	Collector	Sampling site	Data type	LSC length	IR length	SSC length	PCG	rRNA	tRNA
*Amaranthus tricolor*	*Amaranthus tricolor*	OM419215	OM419215	150718		–	–	Public data	83890	23998	18832	88	8	37
*Salsola abrotanoides*	*Salsola abrotanoides*	MW123092	MW123092	151622		–	–	Public data	83737	24638	18609	85	8	37
*Salsola abrotanoides*	*Salsola abrotanoides*	ON149856	ON149856	151613		–	–	Public data	84646	23701	19565	85	8	37
*Salsola affinis*	*Salsola affinis*	ON080842	ON080842	151722		–	–	Public data	83562	24707	18746	86	8	37
*Salsola affinis*	*Salsola affinis*	PP754487	PP754487	151239		–	–	Public data	83324	24585	18745	88	8	37
*Salsola affinis*	*Salsola affinis*	ZJ355	C_AA478711.1	151415	Anren Li	Zhili Suo	Xingjiang, China	This study	83449	24722	18522	85	8	37
*Salsola affinis*	*Salsola affinis*	ZJ356	C_AA478712.1	151402	Anren Li	Zhili Suo	Xingjiang, China	This study	83438	24708	18548	85	8	37
*Salsola arbuscula*	*Salsola arbuscula*	ZJ369	C_AA478725.1	151038	Anren Li	Zhili Suo	Xingjiang, China	This study	84132	23550	19806	84	8	37
*Salsola arbuscula*	*Salsola arbuscula*	ZJ370	C_AA478726.1	151037	Anren Li	Zhili Suo	Xingjiang, China	This study	84131	23550	19806	84	8	37
*Pyankovia brachiata*	*Salsola brachiata*	ON651428	ON651428	149922		–	–	Public data	83445	23874	18729	87	8	37
*Salsola bracchita*	*Salsola bracchita*	ZJ362	C_AA478718.1	149653	Anren Li	Zhili Suo	Xingjiang, China	This study	83459	23954	18286	85	8	37
*Salsola bracchita*	*Salsola bracchita*	ZJ363	C_AA478719.1	149759	Anren Li	Zhili Suo	Shawanqiao, Xinjiang, China	This study	83459	23950	18400	85	8	37
*Salsola collina*	*Salsola collina*	OK189514	OK189514	152923		–	–	Public data	84025	24912	19074	86	8	37
*Salsola collina*	*Salsola collina*	ON149855	ON149855	150585		–	–	Public data	83974	24131	18349	85	8	37
*Salsola collina*	*Salsola collina*	ZJ350	C_AA478707.1	152491	Anren Li	Zhili Suo	Fangshan, Beijing, China	This study	83965	24934	18658	85	8	37
*Salsola collina*	*Salsola collina*	ZJ351	C_AA478708.1	152636	Anren Li	Zhili Suo	Xingjiang, China	This study	83970	24896	18874	85	8	37
*Salsola collina*	*Salsola collina*	ZJ353	C_AA478710.1	152741	Anren Li	Zhili Suo	Minqin, Gansu, China	This study	84009	24952	18828	85	8	37
*Salsola tragus*	*Salsola collina*	ZJ371	C_AA478727.1	152738	Anren Li	Zhili Suo	Xingjiang, China	This study	84010	24952	18824	85	8	37
*Salsola foliosa*	*Salsola foliosa*	PP754489	PP754489	151577		–	–	Public data	83758	24575	18669	88	8	37
*Salsola heptapotamica*	*Salsola heptapotamica*	MZ230595	MZ230595	150738		–	–	Public data	84092	24121	18404	88	8	37
*Salsola implicata*	*Salsola implicata*	ZJ365	C_AA478721.1	150717	Anren Li	Zhili Suo	Xingjiang, China	This study	82915	24648	18506	85	8	37
*Salsola implicata*	*Salsola implicata*	ZJ364	C_AA478720.1	150716	Anren Li	Zhili Suo	Ganjiahu, Xinjiang, China	This study	82914	24648	18506	85	8	37
*Salsola micranthera*	*Salsola micranthera*	ZJ366	C_AA478722.1	150748	Anren Li	Zhili Suo	Minqin, Gansu, China	This study	83527	24220	18781	84	8	37
*Salsola micranthera*	*Salsola micranthera*	ZJ368	C_AA478724.1	150851	Anren Li	Zhili Suo	Xingjiang, China	This study	83527	24220	18884	84	8	37
*Caroxylon nitrarium*	*Salsola nitrarium*	OR552116	OR552116	150777		–	–	Public data	83214	24515	18533	88	8	38
*Caroxylon orientale*	*Salsola orientale*	OR551471	OR551471	151307		–	–	Public data	83685	24500	18622	88	8	37
*Salsola orientale*	*Salsola orientale*	ZJ367	C_AA478723.1	150967	Anren Li	Zhili Suo	Xingjiang, China	This study	83676	24239	18813	84	8	37
*Salsola passerinum*	*Salsola passerinum*	MW192441	MW192441	150925		–	–	Public data	82823	24643	18816	88	8	35
*Salsola pellucida*	*Salsola pellucida*	ZJ352	C_AA478709.1	152612	Anren Li	Zhili Suo	Tulufan, Xinjiang, China	This study	84106	24935	18636	85	8	37
*Salsola pellucida*	*Salsola pellucida*	ZJ361	C_AA478717.1	152612	Anren Li	Zhili Suo	Tulufan, Xinjiang, China	This study	84106	24935	18636	84	8	37
*Kali pellucidum*	*Salsola pellucida*	PP073953	PP073953	152612		–	–	Public data	84106	24935	18636	84	8	37
*Salsola sinkiangensis*	*Salsola sinkiangensis*	PP708569	PP708569	152621		–	–	Public data	84011	24990	18630	86	8	37
*Salsola sinkiangensis*	*Salsola sinkiangensis*	ZJ357	C_AA478713.1	152621	Anren Li	Zhili Suo	Xingjiang, China	This study	84011	24990	18630	84	8	37
*Salsola subcrassa*	*Salsola subcrassa*	ZJ358	C_AA478714.1	150242	Anren Li	Zhili Suo	Fukang, Xinjiang, China	This study	83010	24405	18422	83	8	37
*Salsola subcrassa*	*Salsola subcrassa*	ZJ359	C_AA478715.1	105340	Anren Li	Zhili Suo	Fukang, Xinjiang, China	This study	–	–	–	–	–	–
*Salsola subcrassa*	*Salsola subcrassa*	ZJ360	C_AA478716.1	149892	Anren Li	Zhili Suo	Fukang, Xinjiang, China	This study	84445	23433	18581	83	8	37
*Salsola tragus*	*Salsola tragus*	PP754490	PP754490	152969		–	–	Public data	83985	24909	19166	88	8	36
*Salsola tragus*	*Salsola tragus*	ZJ372	C_AA478728.1	151752	Anren Li	Zhili Suo	Wucaiwan, Xinjiang, China	This study	84994	23372	20014	83	8	37
*Salsola tragus*	*Salsola tragus*	ZJ373	C_AA478729.1	152582	Anren Li	Zhili Suo	Yuzhong, Lanzhou, Gansu, China	This study	83984	24901	18796	85	8	37
*Salsola zaidamica*	*Salsola zaidamica*	ON149859	ON149859	152933		–	–	Public data	84015	25169	18580	87	8	37
*Salsola monoptera*	*Salsola collina*	PP234571	PP234571	152828		–	–	Public data	84079	25124	18501	87	8	37

### 2.2 Acquisition of plastid whole genome sequences

In this study, total genomic DNA was extracted using the modified CTAB (mCTAB) method (Li et al., 2013). Isolated DNA, ranging from 1.0 to 3.0 µg, was utilized to construct a 350 bp paired-end sequencing library. Using an Illumina HiSeq X Ten next-generation sequencing platform in paired-end 150 (PE150) mode, we generated over two gigabytes (Gb) of data was generated. After assembly, the complete sequence of the plastome was obtained. Clean reads obtained from SPAdes software were assembled into contigs (Bankevich et al., 2012). Subsequently, BLAST software was employed to align these contigs against the reference sequence *Salsola affinis* ON080842. A more permissive reference sequence was selected to filter out contigs belonging to the plastome (Ye et al., 2006). The contigs related to the plastome were then connected based on their overlapping regions using Sequencher v5.4.5. After acquiring the complete plastome sequence, the tools Plann and Geneious were used for comprehensive annotation of the *Salsola* plastome sequences (Huang and Cronk, 2015). To ensure the accuracy of the annotation results, Sequin software was also employed for meticulous review and necessary corrections. Finally, to visually present the characteristics of the plastome, the online program OGDraw was used to generate a circular map of the complete plastome (http://chlorobox.mpimp-golm.mpg.de/OGDraw.html).

### 2.3 Boundary and global comparison comparative analysis of the plastomes

In this study, utilizing the online tool IRscope (https://irscope.shinyapps.io/irapp/), we performed an in-depth visual analysis of the annotated plastome map. The primary objective of this step was to investigate and compare the differences in the size and distribution of genes within the boundary region of the plastome, thereby further elucidating its structural characteristics. We conducted a global comparative analysis of the plastomes from 14 species (a total of 23 samples) using the genome online software mVISTA, employing the Shuffle-LAGAN analysis model. *Salsola tragus* was chosen as the reference genome, and the global genome differences were visualized to emphasize sequence fragments with high variability (Frazer et al., 2004).

### 2.4 Comparative analysis of repeated sequences and indels of *Sasola* plastomes

In the plastome, repeat sequences are a crucial component of the gene regulatory network, appearing at the same or different positions in the form of repeat unit fragments. This study employed REPuter to precisely locate forward repeat sequences, reverse repeat sequences, palindromic repeat sequences, and complementary repeat sequences within the genome (Kurtz et al., 2001). We also used the GMATA tool to predict simple sequence repeats (SSRs) (Wang and Wang, 2016). When configuring SSR parameters, we established that if the repeat unit was a single base, it must be repeated at least ten times; if the unit consisted of two bases, it must be repeated at least five times; a three-base unit must be repeated at least four times; and for four-base, five-base, and six-base units, the units had to be repeated at least three times. After determining the thresholds and repetition counts for these parameters, we meticulously manually calibrated all SSR predictions to eliminate unnecessary redundancies and ensure data accuracy.

### 2.5 Nucleotide polymorphism and codon usage bias analysis of plastomes

To assess nucleotide polymorphism in both the protein-coding and non-coding regions of the plastome of *Salsola* species, this study employed DnaSP v6 software in conjunction with sliding window analysis (Rozas et al., 2017). The window length was set to 600 bp, and the step size was set to 200 bp. After performing the calculations, we visualized and presented the results as line graphs. Subsequently, we conducted an in-depth analysis of specific fragments with high polymorphism within the plastome of the *Salsola* genus. CodonW was utilized to evaluate the patterns of synonymous codon usage in protein-coding genes (https://codonw.sourceforge.net/). Codon usage bias was quantified using the Relative Synonymous Codon Usage (RSCU) index, which represents the ratio of the observed frequency of a codon to the expected frequency under the assumption of equal usage of synonymous codons. Codons with RSCU values greater than 1.0 were deemed to be preferentially used.

### 2.6 Phylogenetic analysis

Although plastome sequences are generally highly conserved, sequence variation still provides abundant phylogenetically informative characters for resolving evolutionary relationships. In this study, we carried out a detailed phylogenetic analysis of the obtained sequences of the *Salsola* genus, aiming to uncover the phylogenetic relationships among these species through comprehensive data. For sequence alignment, we selected MAFFT v7.409 software to ensure the accuracy of the alignment results. Subsequently, a phylogenetic tree was constructed using the complete plastome sequences, with the objective of conducting an in-depth study of the evolutionary relationships among these species (Katoh and Standley, 2013). The maximum likelihood (ML) method and Bayesian inference (BI) were implemented using PhyloSuite software (Mau et al., 1999; Kannan and Wheeler, 2012; Xiang et al., 2023). The best-fit models for maximum-likelihood (ML) analyses were selected according to the Bayesian information criterion (BIC) by ModelFinder (Kalyaanamoorthy et al., 2017). The ML method was used to construct a phylogenetic tree. ML analyses were performed using IQ-TREE v.1.6.8 (Minh et al., 2020), with the GTR+F+I model and 1000 standard bootstrap replicates. MrBayes v.3.2.6 was used to construct phylogeny tree, with the GTR+F+I model (Ronquist et al., 2012). Bayesian analyses were conducted using four concurrent Markov chain Monte Carlo (MCMC) chains for 1,000,000 generations, with tree topologies recorded at intervals of 1,000 generations. The phylogenetic trees and branch support values were visualized using FigTree v.1.4.2.

## 3 Results

### 3.1 Comparative analysis of *Salsola* plastomes

**Figure 1:**
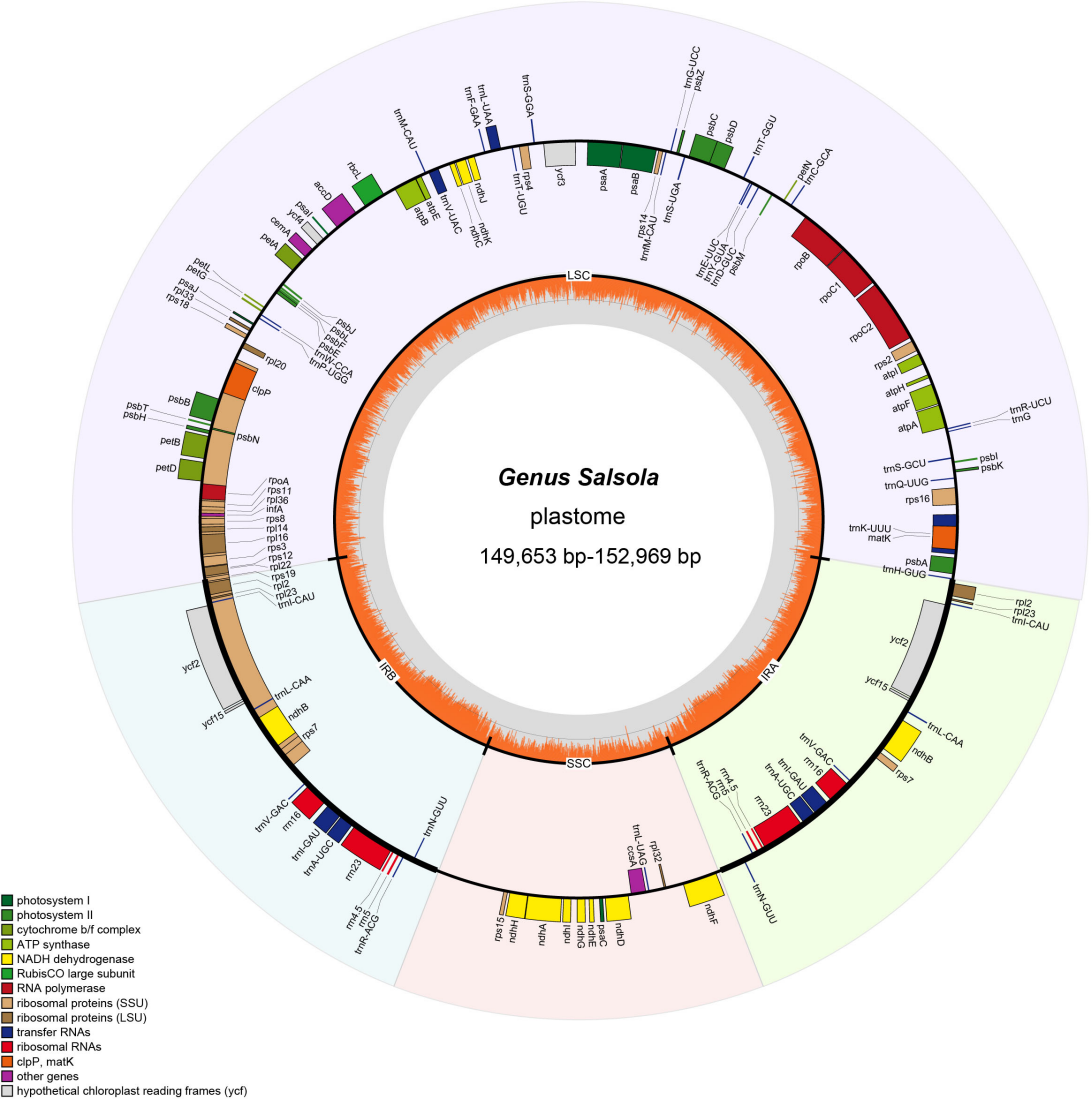
Plastome map of *Salsola*. Legend (top to bottom): photosystem I, photosystem II, cytochrome b6/f complex, ATP synthase, NADH dehydrogenase, RubisCO large subunit, RNA polymerase, ribosomal proteins (SSU/LSU), transfer RNA, ribosomal RNA, *clpP* and *matK*, other genes, and hypothetical chloroplast reading frame (*ycf*).

After the sequencing process, we successfully obtained at least two Gb of clean data for each sample. We successfully assembled and curated the complete plastome sequences from 23 samples representing 14 species. Subsequent functional annotation provided a comprehensive genomic profile for each species. The plastomes of the studied *Salsola* species exhibit a highly conserved quadripartite structure. As illustrated in **Figure 1**, the circular DNA molecule comprises a Large Single-Copy (LSC) region, a Small Single-Copy (SSC) region, and two identical Inverted Repeats (IRa and IRb). Across the *Salsola* species, total plastome lengths range from 149,653 bp in *S. brachiata* to 152,969 bp in *S. tragus* (**Table 1**).

### 3.2 Sequence similarity analysis of *Salsola* plastomes

**Figure 2:**
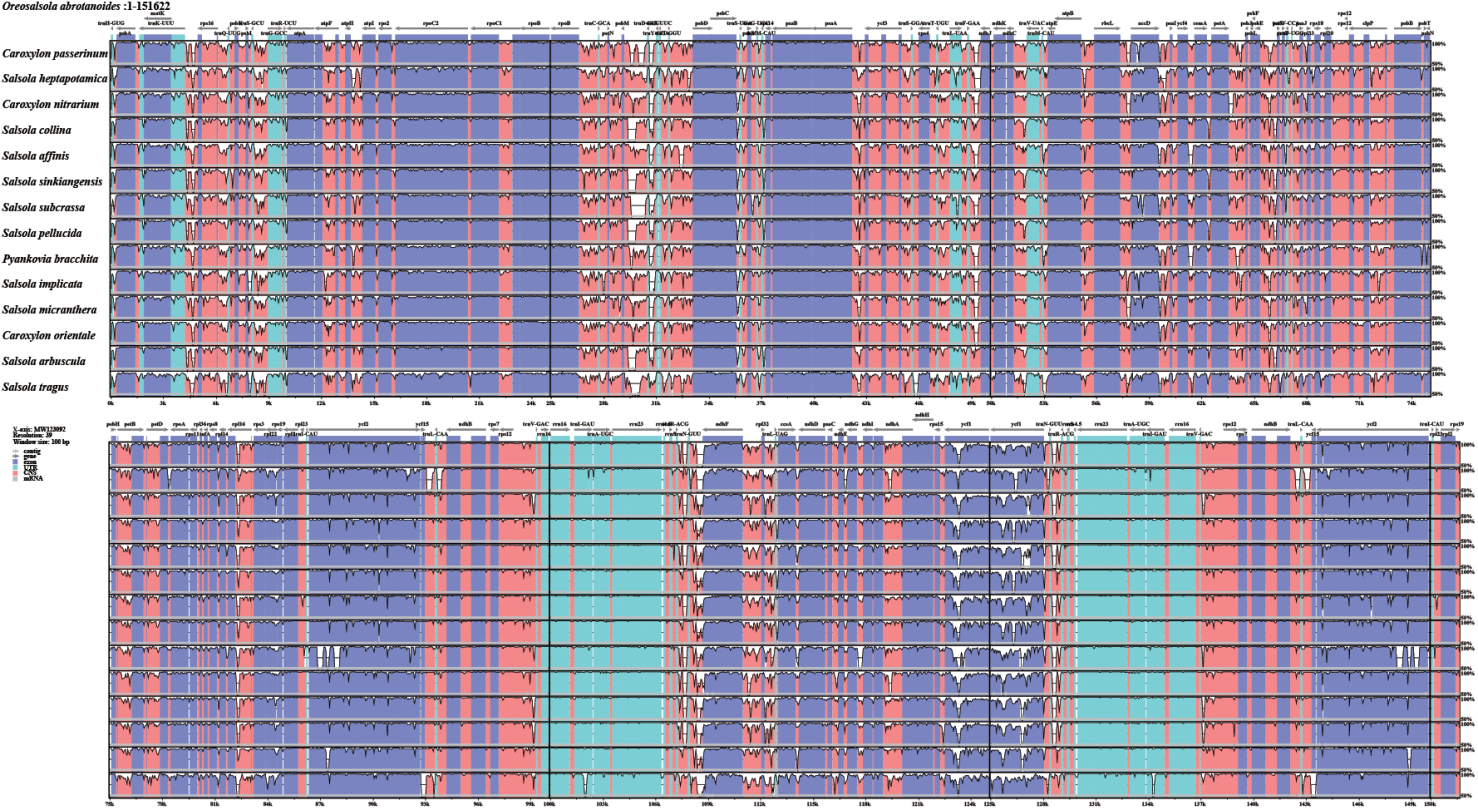
Visualization and comparison of plastome sequences using *Salsola abrotanoides* as the reference.

To comprehensively characterize sequence variation and microsatellite features in the chloroplast genomes, we analyzed nucleotide polymorphism and the distribution patterns of simple sequence repeats (SSRs) across different genomic regions. Overall, the plastomes exhibited a high level of sequence conservation, whereas SSRs were unevenly distributed among genomic regions, with the LSC region containing the highest proportion of repeats (**Figure 2**). Nucleotide polymorphism varied across different genomic positions. The LSC region exhibited minimal nucleotide polymorphism, indicating relative stability. In contrast, the SSC region showed more pronounced fluctuations, though overall divergence remained low.

**Figure 3:**
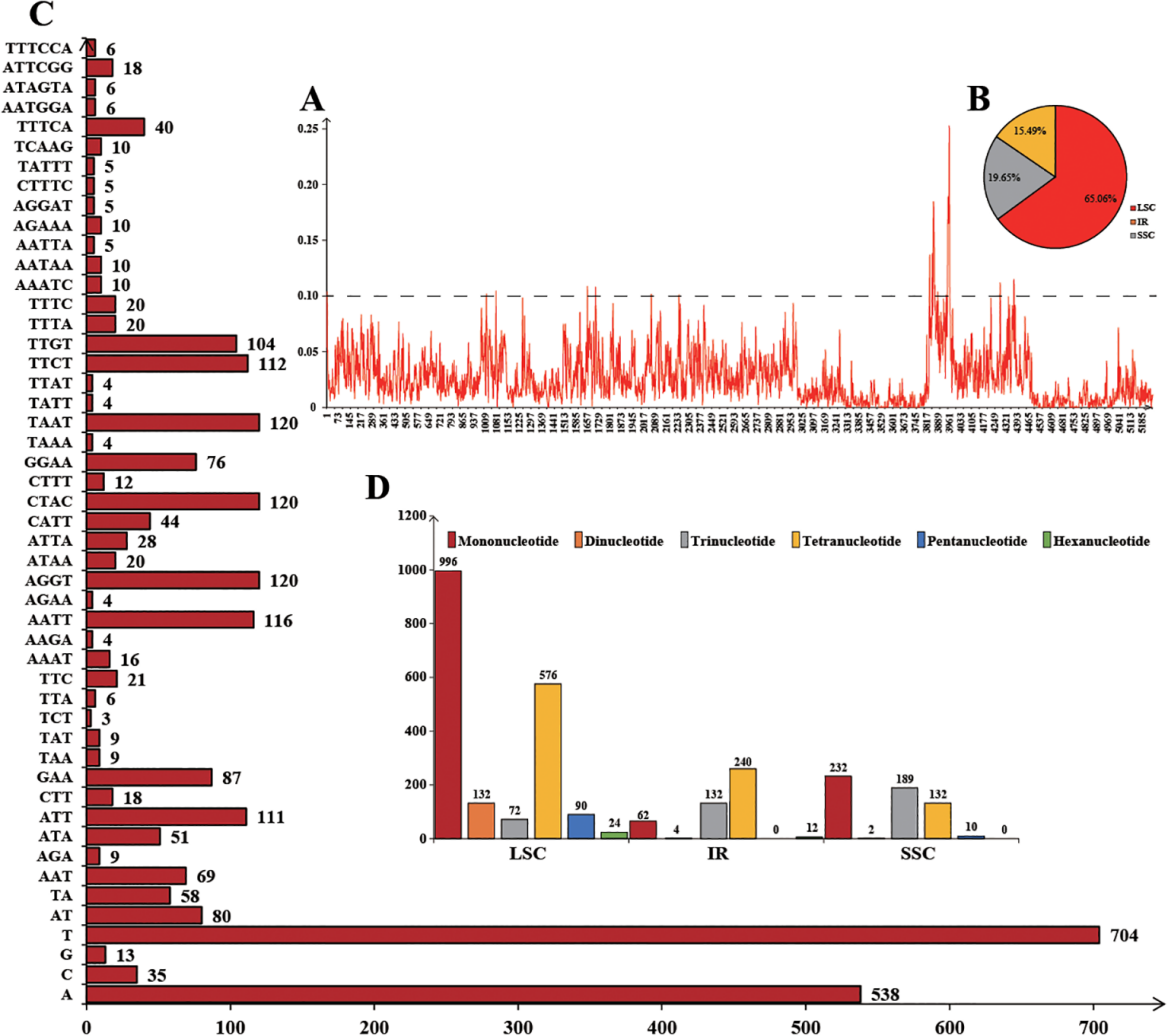
Variation analyses of *Salsola* plastomes. **(A)** Nucleotide diversity (Pi) analysis across the complete plastomes of sampled *Salsola* species. The sliding-window analysis was performed to evaluate nucleotide diversity along the plastome, with nucleotide diversity (Pi) plotted against genomic positions. The distribution of polymorphic sites revealed differences in sequence variability among the LSC, SSC, and IR regions. **(B)** Regional distribution of simple sequence repeats (SSRs) in *Salsola* plastomes. The proportions of SSR loci located in the LSC, SSC, and IR regions are shown, indicating the uneven distribution of SSRs among different genomic regions. **(C)** Frequency distribution of different SSR motif types identified in *Salsola* plastomes. Mononucleotide repeats represent the most abundant SSR category, followed by dinucleotide, trinucleotide, tetranucleotide, pentanucleotide, and hexanucleotide repeats. The numbers at the end of each bar indicate the number of identified SSR loci for each motif type. **(D)** Distribution patterns of different SSR types across the LSC, IR, and SSC regions. The histogram illustrates the abundance of mono-, di-, tri-, tetra-, penta-, and hexanucleotide repeats in each plastome region, highlighting the regional variation in SSR composition.

The IRa and IRb regions demonstrated strong conservation with few highly variable sites and exhibited characteristic symmetry (**Figure 3A**). SSR distribution varied across the four regions: LSC (65.06%), SSC (19.45%), IR (15.49%) (**Figure 3B**). SSR analysis identified various motif types. Mononucleotide repeats (A, T, C, G) were the most abundant, with A/T motifs significantly outnumbering C/G. Dinucleotide repeats consisted of TA (58 loci) and AT (80 loci). Additionally, the genomes contained 11 trinucleotide, 19 tetranucleotide, and nine pentanucleotide loci. Four hexanucleotide motifs were identified (TTTCCA, ATTCGG, ATAGTA, AATGGA); except for ATTCGG (18 loci), the remaining motifs each appeared six times (**Figure 3C**). Across regions, the LSC harbored the highest frequency of most SSR types, while the IR regions consistently showed the lowest density (**Figure 3D**).

To further assess structural variation among plastomes, insertion/deletion (indel) events were identified and compared across the LSC, SSC, IRa, and IRb regions of all sampled taxa. Overall, indels were unevenly distributed among the four genomic regions, with the LSC region consistently containing the highest number of indels, whereas the IR regions exhibited relatively few events, reflecting their highly conserved nature. Variation in indel abundance among species was observed primarily in the single-copy regions, particularly the LSC. The indel counts in the LSC, IRb, SSC, and IRa regions for each species are as follows: *Caroxylon nitrarium* OR552116 (45, 5, 13, 9); *Caroxylon orientale* OR551471 (52, 4, 17, 8); *Caroxylon passerinum* MW192441 (56, 4, 14, 9); *Pyankovia brachiata* ON651428 (51, 7, 22, 11); *Oreosalsola abrotanoides* MW123092 (70, 5, 16, 5); *Salsola affinis* ON080842 (71, 6, 13, 9); *S. collina* OK189514 (76, 4, 22, 13); *S. heptapotamica* MZ230595 (63, 10, 9, 10); *S. collina* ZJ350 (69, 4, 29, 7); *S. collina* ZJ351 (70, 4, 25, 16); *S. pellucida* ZJ352 (74, 4, 23, 7); *S. collina* ZJ353 (71, 4, 22, 16); *S. affinis* ZJ355 (70, 7, 12, 10); *S. affinis* ZJ356 (70, 6, 12, 9); *S. sinkiangensis* ZJ357 (71, 4, 25, 7); *S. subcrassa* ZJ358 (66, 5, 21, 15); *S. subcrassa* ZJ359 (62, 0, 15, 0); *S. subcrassa* ZJ360 (65, 5, 13, 8); *S. pellucida* ZJ361 (74, 4, 23, 7); *S. brachiata* ZJ362 (49, 7, 19, 11); *S. brachiata* ZJ363 (same as ZJ362); *S. implicata* ZJ364 (37, 4, 19, 11); *S. implicata* ZJ365 (36, 4, 19, 11); *S. micranthera* ZJ366 (49, 5, 12, 9); *Caroxylon orientale* ZJ367 (53, 4, 25, 11); *Salsola micranthera* ZJ368 (49, 5, 13, 8); *S. arbuscula* ZJ369 (68, 4, 20, 9); *S. arbuscula* ZJ370 (same as ZJ369); *S. tragus* ZJ371 (71, 4, 22, 16); *S. tragus* ZJ372 (49, 6, 9, 6); *S. tragus* ZJ373 (66, 5, 22, 11).

### 3.3 Comparative analysis of plastome boundaries in *Salsola* species

**Figure 4:**
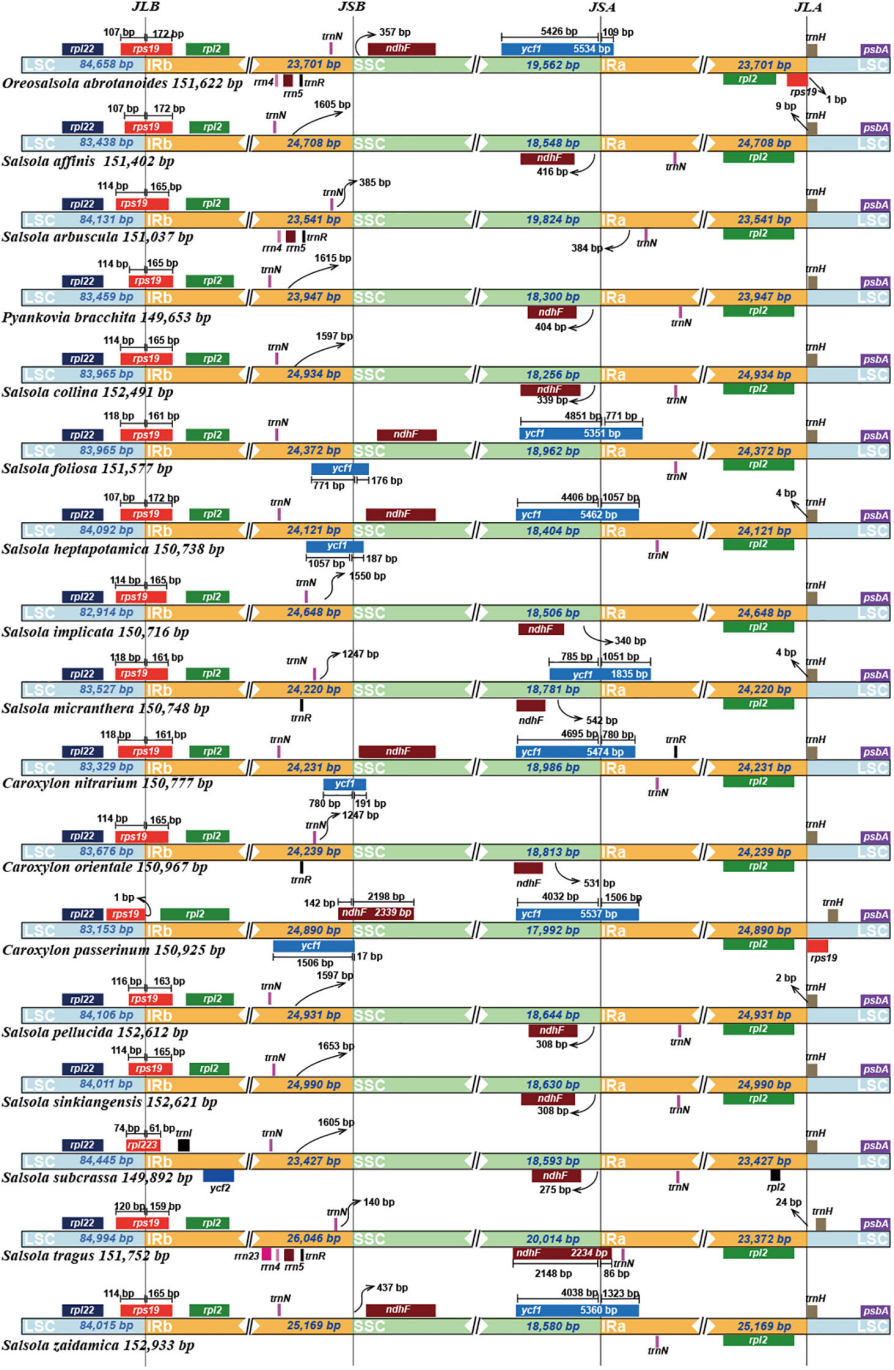
Comparison of the LSC, SSC, and IR boundary regions among plastomes of *Salsola* species.

To investigate structural variation in plastomes, the junctions between the LSC, SSC, and IR regions were compared across the *Salsola* species. Overall, the boundaries were highly conserved, with only minor expansions and contractions of the IR regions observed among species. Most differences were associated with the positioning of genes adjacent to the junctions rather than large-scale structural rearrangements. In the LSC region, all species contained *rpl22*, *trnH*, and *psbA*. While *rps19* spanned the LSC/IRb boundary in most species, it was located entirely within the LSC in *C. passerinum*; conversely, *rpl22* spanned the LSC/IRb boundary only in *S. subcrassa*. Within the IRb region, *C. passerinum* uniquely lacked *trnN* but possessed a partial *ndhF* fragment, while *S. subcrassa* contained complete copies of *trnL* and *ycf2*. In the SSC region, partial *ycf1* fragments were identified in *C. passerinum*, *S. heptapotamica*, *C. nitrarium*, *S. abrotanoides*, *S. tragus*, *Salsola zaidamica* and *S. micranthera*. Notable IRa region features included the exclusive presence of *rpl2* in *S. subcrassa*, *rps19* in *S. abrotanoides*, and *trnR* in *C. nitrarium* (**Figure 4**).

### 3.4 Codon usage bias in the genus *Salsola*

**Figure 5:**
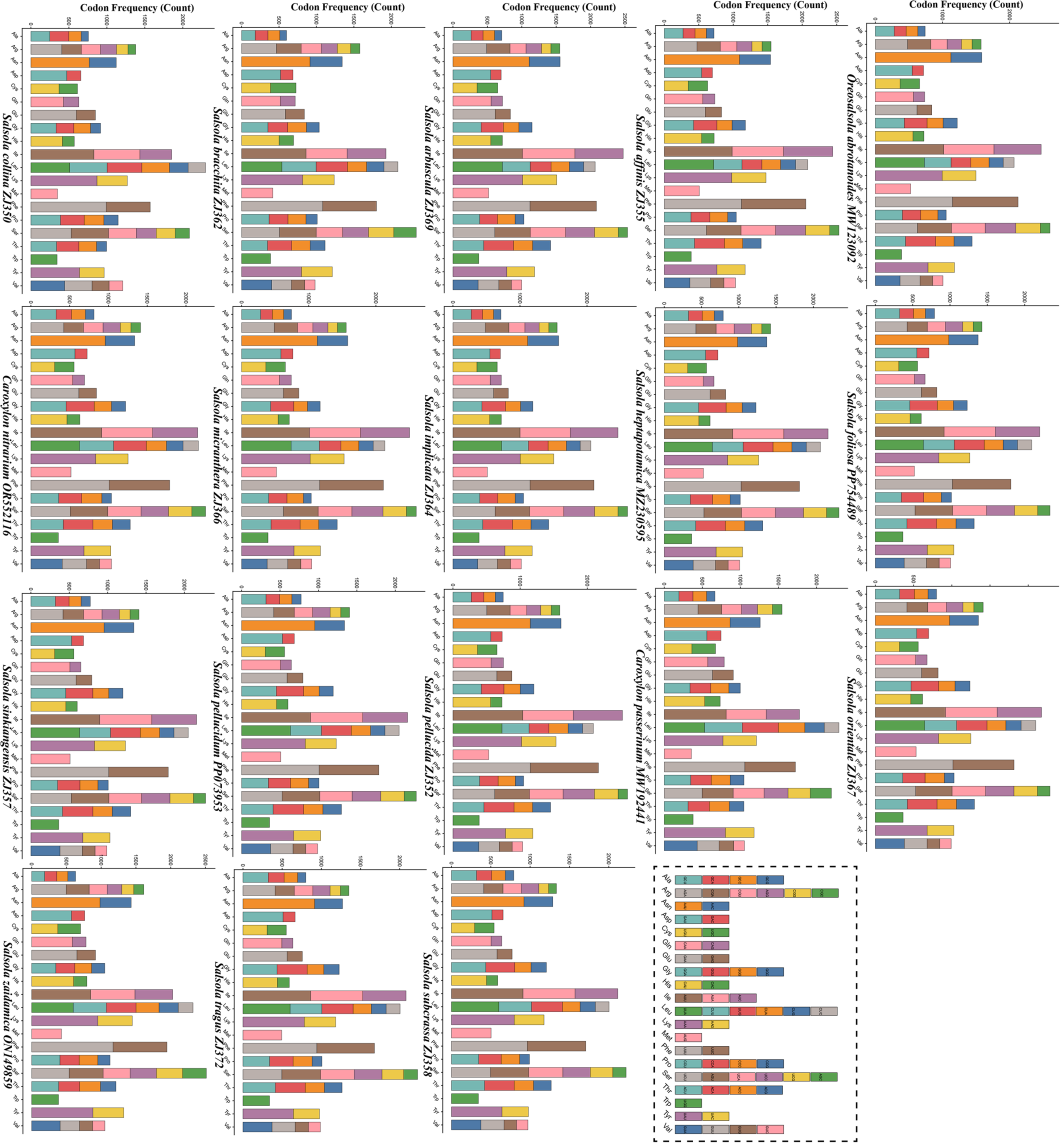
Codon usage bias across *Salsola* species. Each species is presented in an individual panel, with the species name indicated on the left side. The legend is positioned in the lower right corner of the figure.

To evaluate codon usage variation among *Salsola* plastomes, codon composition and synonymous codon usage patterns were compared across all species. The results revealed a highly conserved codon usage profile, indicating limited variation in coding sequence evolution within the genus. Codon usage bias across the genus *Salsola* is generally consistent and aligns with typical patterns observed in angiosperms. Specifically, there is a clear preference for codons ending in A/U (A/T in DNA), reflecting a weak to moderate bias primarily driven by mutational pressure and natural selection--a common feature in flowering plants (**Figure 5**).

### 3.5 Phylogenetic analysis of *Salsola* species

**Figure 6:**
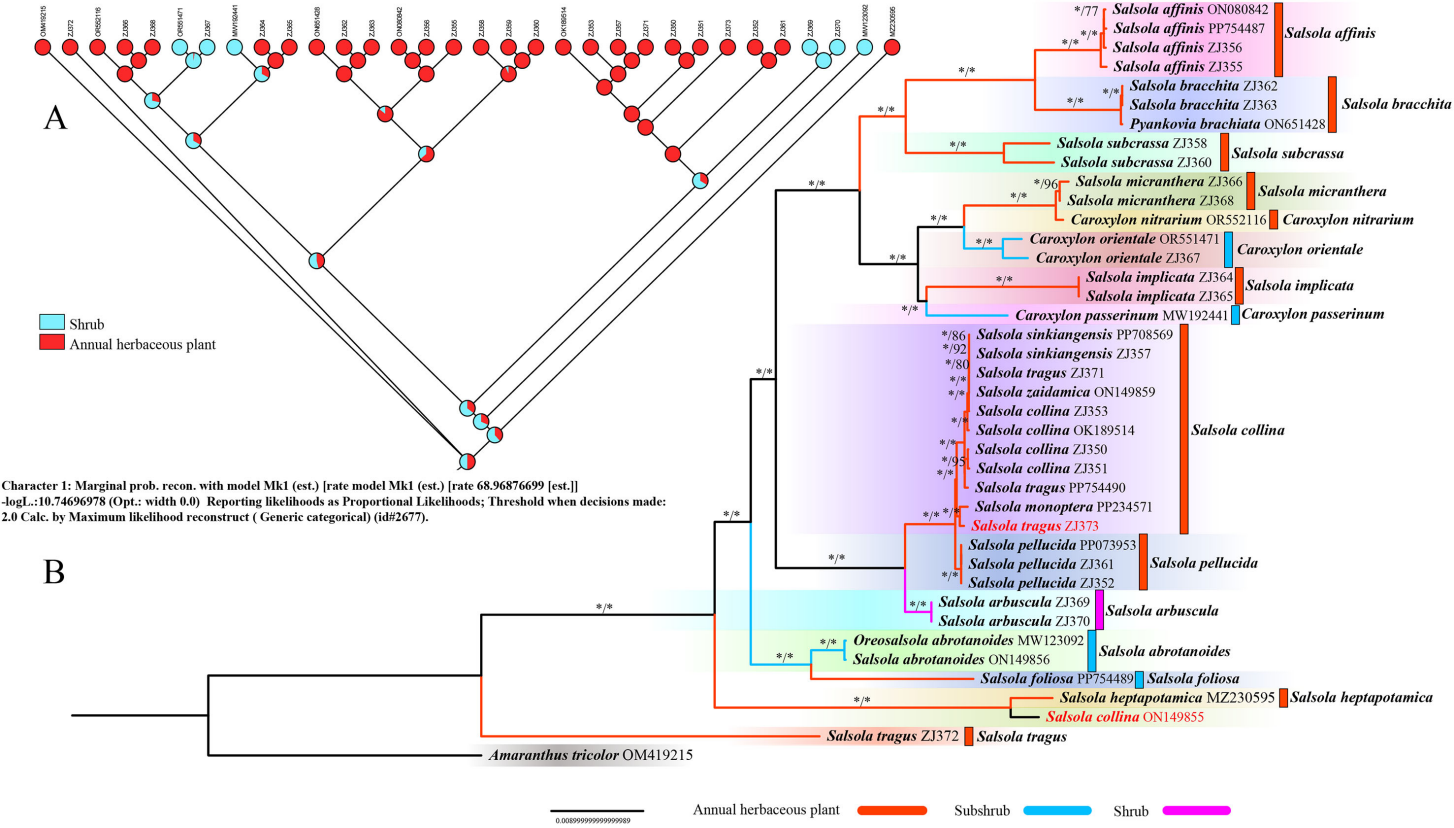
Phylogenetic relationships of *Salsola* species and related taxa based on complete plastome sequences. **(A)** Phylogenetic tree with growth forms indicated for the sampled taxa, showing the distribution of different life forms across the inferred lineages. **(B)** Comparison of phylogenetic trees reconstructed using Bayesian inference (BI) and maximum likelihood (ML) methods. Numbers at nodes indicate Bayesian posterior probabilities (BPP) and bootstrap support values (BS) from BI and ML analyses, respectively. Asterisks (*) indicate nodes with maximum support values (BPP = 1.00 or BS = 100%).

To clarify the phylogenetic relationships among members of *Salsola* and its allied taxa, phylogenetic analyses were conducted using complete plastome sequences. The ML and BI phylogenetic trees showed identical topological structures, with most nodes receiving strong support values. In addition, the phylogenetic tree constructed based on growth forms exhibited the same overall topology as the BI and ML trees, with consistent relationships among the sampled taxa (**Figure 6**). All sampled taxa were grouped into a strongly supported *Salsola* lineage, within which several distinct species-level clades were recovered. Species previously assigned to *Caroxylon* and *Pyankovia* were resolved within the *Salsola* clade rather than forming separate lineages. The sampled *Caroxylon* species were distributed among different branches of the *Salsola* clade, while *Pyankovia brachiata* was clustered together with *Salsola* species. Hence, phylogenetic analysis based on the reconstructed tree suggests that plastome evidence is consistent with the inclusion of *Caroxylon*, *Oreosalsola*, and *Pyankovia* within *Salsola* sensu lato. In addition, the phylogenetic tree revealed that *S. tragus* and *S. heptapotamica* form a sister clade, which is subsequently sister to *S. abrotanoides*. The latter is sister to the clade comprising *S. arbuscula* and *S. pellucida*. *S. collina* clusters closely with *S. pellucida*, together forming a distinct lineage with *Caroxylon passerinum*. Additionally, *Caroxylon orientale* groups with the clade containing *Caroxylon nitrarium* and *S. micranthera*. Finally, *S. subcrassa* is sister to the *S. affinis*-*S. brachiata* pair (**Figure 6B**).

The phylogenetic tree also revealed clear differentiation among the sampled *Salsola* species with different growth forms. Shrub and subshrub species were distributed in different positions within the *Salsola* clade, while most annual herbaceous species were grouped into several well-supported lineages. Overall, the plastome-based phylogeny provided a highly supported framework for resolving relationships among *Salsola* species and related taxa. More specifically, the *Salsola* samples are divided into two basal clades. Annual herbs constitute the majority, including *S. brachiata*, *S. affinis*, *S. subcrassa*, *S. micrantherum*, *Caroxylon nitrarium*, *S. implicata*, *S. collina*, *S. pellucida*, *S. heptapotamica*, and *S. tragus*. In contrast, *S. arbuscula* is the sole shrub, while *Caroxylon orientalis*, *Caroxylon passerinum*, and *Oreosalsola abrotanoides* are classified as subshrubs (**Figure 6A**).

## 4 Discussion

### 4.1 Plastome genome conservation and structural stability in *Salsola*

The plastomes of *Salsola* species exhibit a highly conserved quadripartite structure composed of LSC region, SSC region, and two inverted repeats (IRa/IRb), with genome sizes ranging from 149,653 to 152,969 bp. Across analyzed species, no major structural rearrangements or gene losses were detected, indicating strong structural conservation consistent with typical angiosperm chloroplast genomes (Zuntini et al., 2024). This high level of conservation suggests that plastome architecture in *Salsola* has remained relatively stable during long-term evolutionary history. In particular, the IR regions display remarkable sequence conservation and reduced nucleotide variability compared with the single-copy regions. This stability is generally attributed to the presence of duplicated IR regions, which facilitate gene conversion and copy-dependent repair mechanisms that efficiently eliminate deleterious mutations, thereby reducing substitution rates (Hase et al., 2006; Li et al., 2016). In addition, homologous recombination between IR copies further enhances sequence homogenization. Previous studies have demonstrated that IR regions evolve significantly more slowly than LSC and SSC regions, with synonymous substitution rates typically reduced to one-third or even one-quarter of those observed in single-copy regions (Wolfe et al., 1987; Zhu et al., 2016). The present results in *Salsola* are fully consistent with this evolutionary pattern, further supporting the concept of the IR region as a “low-evolutionary-rate buffer” within the plastome.

### 4.2 Potential molecular markers for species identification

Despite the overall structural conservation of the plastomes, *Salsola* species exhibit localized sequence variation, particularly in the SSC region and parts of the LSC region. These variations are mainly represented by single nucleotide polymorphisms (SNPs), simple sequence repeats (SSRs), and insertion–deletion (indel) events, providing abundant sequence variation for comparative genomic and phylogenetic analyses. SSR analysis revealed a predominance of mononucleotide repeats, especially A/T-rich motifs, which is consistent with the AT-rich composition commonly reported in angiosperm plastomes. SSRs are generally considered to arise through replication slippage and have been widely used as informative molecular markers because of their relatively high mutation rates and intraspecific polymorphism (Provan et al., 2001). In this study, SSRs were most abundant in the LSC region and least frequent in the IR regions, further reflecting the stabilizing effect of IR regions-mediated repair mechanisms. In addition, indel events showed clear interspecific variation and were particularly enriched in single-copy regions. Together with SSRs, these highly variable regions provide valuable molecular resources for species identification in *Salsola*. This is particularly relevant for morphologically similar taxa, where traditional morphological characters alone may be insufficient for accurate species identification. Compared with conventional plastid DNA barcodes (e.g., *rbcL* and *matK*), complete plastome sequences contain substantially more phylogenetically informative characters and therefore have the potential to improve species discrimination. However, the performance of the identified hotspot regions should be further evaluated through comparisons with commonly used plastid DNA barcodes (Li et al., 2015). Therefore, the observed variation patterns not only enhance our understanding of plastome evolution but also provide practical molecular resources for accurate species delimitation.

### 4.3 Phylogenetic relationships and taxonomic implications within the *Salsola* complex

The phylogenetic relationships reconstructed in this study showed a high level of consistency among different analytical approaches. Both Bayesian inference (BI) and maximum likelihood (ML) analyses based on complete plastome sequences recovered identical topologies, with strong statistical support for most nodes, indicating the robustness and reliability of the inferred relationships among the sampled taxa. In addition, the phylogenetic tree reconstructed after incorporating growth form information recovered the same overall topology as the plastome-based BI and ML trees, further supporting the stability of the inferred evolutionary relationships. These results indicate that complete plastome sequences provide sufficient phylogenetic information to resolve relationships among closely related members of the *Salsola* complex. The distribution of growth forms also showed a certain phylogenetic pattern. Among the sampled taxa, annual herbs and subshrubs were mainly represented within *Salsola* sensu stricto, whereas shrub species were primarily associated with other sampled lineages traditionally included in *Salsola* sensu lato. Nevertheless, similar growth forms were observed in multiple phylogenetic lineages, suggesting that growth habit alone may not accurately reflect phylogenetic relationships within the *Salsola* complex. Therefore, plastome-scale molecular evidence should be interpreted together with morphological and ecological characters when evaluating evolutionary relationships among closely related taxa.

Our phylogenetic reconstruction is generally consistent with previous molecular systematic studies of *Salsoleae*. Wen et al. (2010), based on plastid DNA fragments and the nuclear ITS region, reported that the traditionally circumscribed *Salsola* sensu lato was not recovered as a monophyletic group but instead comprised two major evolutionary lineages. One lineage was closely associated with *Anabasis*, *Halogeton*, *Haloxylon*, *Girgensohnia*, *Iljinia*, *Horaninowia*, and *Sympegma*, whereas the other clustered with *Nanophyton*, *Petrosimonia*, *Ofaiston*, *Climacoptera*, and *Halimocnemis*. Likewise, phylogenetic analyses based on plastome data have provided a well-resolved framework for understanding evolutionary relationships within the *Salsola* complex in Amaranthaceae s.l. under the APG IV classification system (The Angiosperm Phylogeny Group et al., 2016). Compared with analyses based on a limited number of plastid markers, complete plastome sequences substantially improved phylogenetic resolution and statistical support for relationships among the sampled taxa, consistent with previous studies demonstrating the advantages of plastome-scale datasets for resolving relationships among closely related plant species (The Angiosperm Phylogeny Group et al., 2016).

Within the present sampling, *Caroxylon nitrarium* and the sampled plastome sequences of *Pyankovia* were recovered within the broader *Salsola* lineage rather than forming isolated lineages. These relationships are generally consistent with previous phylogenetic hypotheses suggesting close evolutionary affinities among these taxa (Wen et al., 2010). However, the present analyses included only a limited number of representatives from *Caroxylon*, *Pyankovia*, and related genera. Therefore, our results should be interpreted as evidence for the phylogenetic placement of the sampled taxa rather than as conclusive evidence for genus-level circumscription within the *Salsola* complex. Likewise, although the sampled representatives of *Salsola* sensu stricto formed a well-supported clade in our plastome phylogeny, the present sampling does not permit broader conclusions regarding the monophyly or taxonomic limits of the entire genus.

Complete plastome sequences have been widely used to improve phylogenetic resolution in taxonomically complex plant groups because they provide abundant phylogenetically informative characters and generally outperform analyses based on one or a few molecular markers (Moore et al., 2010). Nevertheless, plastid genomes represent only a single, maternally inherited genomic compartment and may not fully reflect the evolutionary history of species in groups affected by hybridization, introgression, or incomplete lineage sorting. Furthermore, the present study sampled only part of the taxonomic diversity within the *Salsola* complex. Therefore, broader taxon sampling, together with evidence from nuclear genomes, morphology, and ecology, will be necessary to further evaluate evolutionary relationships and alternative taxonomic hypotheses within *Salsoleae*.

## 5 Conclusions

This study generated and analyzed complete plastome data from 14 species of the genus *Salsola*, providing new insights into plastome structure, sequence variation, and phylogenetic relationships within this taxonomically complex group. Species of *Salsola* often exhibit convergent morphological traits associated with adaptation to arid and saline environments, which has historically complicated species delimitation and obscured interspecific relationships. The plastomes of *Salsola* species are highly conserved in structure, exhibiting a typical quadripartite organization with genome sizes ranging from 149,653 to 152,969 bp. Despite this overall conservation, comparative analyses revealed localized sequence variation, particularly in non-coding regions, including SSRs and indels, which represent potential mutation hotspots for future molecular marker development. These features provide valuable genetic resources for improving species discrimination within the genus. Phylogenetic analyses based on plastome data resolved major clades within *Salsola* and improved the resolution of interspecific relationships among sampled taxa, thereby providing a robust framework for understanding evolutionary diversification within the genus. Nevertheless, this inference should be viewed with caution, as it is based solely on plastome data and requires further testing with broader taxon sampling and nuclear genomic evidence. Overall, this study expands the plastid genomic resources available for *Salsola* and provides a foundation for future research on phylogeny, taxonomy, species identification and, marker development in this complex genus.

## Data Availability

All plastomes have been uploaded to National Genomics Data Center (NGDC, https://ngdc.cncb.ac.cn/). The accession numbers of the datasets are provided in **Table 1**.
